# Focal cryotherapy: step by step technique description

**DOI:** 10.1590/S1677-5538.IBJU.2016.0664

**Published:** 2017

**Authors:** Cristina Redondo, Victor Srougi, José Batista da Costa, Mohammed Baghdad, Guillermo Velilla, Igor Nunes-Silva, Sebastien Bergerat, Silvia Garcia-Barreras, François Rozet, Alexandre Ingels, Marc Galiano, Rafael Sanchez-Salas, Eric Barret, Xavier Cathelineau

**Affiliations:** 1 Hospital Universitario de Getafe - Servicio de Urología, Getafe, Spain; 2 Hospital das Clínicas da Faculdade de Medicina da USP - Urologia, São Paulo, Brasil; 3 Institut Mutualiste Montsouris Ringgold standard institution, Paris, Île-de-France, France; 4 L’Institut Mutualiste Montsouris - Urology, Paris, France

## Abstract

**Introduction and objective:**

Focal cryotherapy emerged as an efficient option to treat favorable and localized prostate cancer (PCa). The purpose of this video is to describe the procedure step by step.

**Materials and methods:**

We present the case of a 68 year-old man with localized PCa in the anterior aspect of the prostate.

**Results:**

The procedure is performed under general anesthesia, with the patient in lithotomy position. Briefly, the equipment utilized includes the cryotherapy console coupled with an ultrasound system, argon and helium gas bottles, cryoprobes, temperature probes and an urethral warming catheter. The procedure starts with a real-time trans-rectal prostate ultrasound, which is used to outline the prostate, the urethra and the rectal wall. The cryoprobes are pretested and placed in to the prostate through the perineum, following a grid template, along with the temperature sensors under ultrasound guidance. A cystoscopy confirms the right positioning of the needles and the urethral warming catheter is installed. Thereafter, the freeze sequence with argon gas is started, achieving extremely low temperatures (-40°C) to induce tumor cell lysis. Sequentially, the thawing cycle is performed using helium gas. This process is repeated one time. Results among several series showed a biochemical disease-free survival between 71-93% at 9-70 month- follow-up, incontinence rates between 0-3.6% and erectile dysfunction between 0-42% (1–5).

**Conclusions:**

Focal cryotherapy is a feasible procedure to treat anterior PCa that may offer minimal morbidity, allowing good cancer control and better functional outcomes when compared to whole-gland treatment.

## ARTICLE INFO

**Available at: http://www.intbrazjurol.com.br/video-section/20160664_Redondo_et_al**

**Int Braz J Urol. 2017; 43 (Video #14): 995-6**
